# Science responses to IUCN Red Listing

**DOI:** 10.7717/peerj.4025

**Published:** 2017-11-14

**Authors:** Ivan Jarić, David L. Roberts, Jörn Gessner, Andrew R. Solow, Franck Courchamp

**Affiliations:** 1 Leibniz-Institute of Freshwater Ecology and Inland Fisheries, Berlin, Germany; 2 Biology Centre of the Czech Academy of Sciences, Institute of Hydrobiology, České Budějovice, Czech Republic; 3 Institute for Multidisciplinary Research, University of Belgrade, Belgrade, Serbia; 4 Durrell Institute of Conservation and Ecology, School of Anthropology and Conservation, University of Kent, Canterbury, United Kingdom; 5 Woods Hole Oceanographic Institution, Woods Hole, USA; 6 Ecologie, Systematique, and Evolution, Univ. Paris-Sud, CNRS, AgroParisTech, Universite Paris-Saclay, Orsay, France

**Keywords:** Data deficient, Critically endangered, IUCN Red List, Endangered species, Extinction risk

## Abstract

The IUCN Red List of Threatened Species is often advocated as a tool to assist decision-making in conservation investment and research focus. It is frequently suggested that research efforts should prioritize species in higher threat categories and those that are Data Deficient (DD). We assessed the linkage between IUCN listing and research effort in DD and Critically Endangered (CR) species, two groups generally advocated as research priorities. The analysis of the change in the research output following species classification indicated a listing effect in DD species, while such effect was observed in only a minority of CR species groups. DD species, while chronically understudied, seem to be recognized as research priorities, while research effort for endangered species appears to be driven by various factors other than the IUCN listing. Optimized conservation research focus would require international science planning efforts, harmonized through international mechanisms and promoted by financial and other incentives.

## Introduction

A challenging problem is deciding how to allocate scarce resources to the conservation of different species (Pimm, 2000; Bottrill et al., 2008; Jachowski & Kesler, 2009). These resources include scientific effort aimed at increasing knowledge about the current status and future prospects of species, and measures that can be taken to improve the latter. In principle, a useful tool for guiding the allocation of scientific effort is the Red List classification system used by the International Union for Conservation of Nature (IUCN; Rodrigues et al., 2006; Brito, 2008; Brooks et al., 2008; De Lima, Bird & Barlow, 2011). Briefly, this system includes a total of nine categories, ranging from ‘not evaluated’ to ‘extinct’, with intermediate categories reflecting both the state of knowledge and level of threat. The goal of this paper is to explore the effect of the classification of a species to these categories on one aspect of scientific effort—namely, publication rate. For concreteness, we focus on the effect on publication rate of assigning a species to two categories—Data Deficient (DD) and Critically Endangered (CR), using the category Least Concern (LC) as a control.

Although our aim is to detect and characterize the effect of IUCN classification on publication rate, it is worth asking what some plausible outcomes might be. Of course, one plausible outcome is that publication rate is simply unresponsive to the classification of a species as either DD or CR. There are, in addition, at least two plausible patterns of response. One is a listing effect in publication rate for species classified as CR that exceeds any effect for species classified as DD. This is consistent with allocating resources toward the most urgent cases (Brito, 2008; Brooks et al., 2008; De Lima, Bird & Barlow, 2011). A second plausible pattern is a listing effect in the publication rate for species classified as DD that exceeds any effect for species classified as CR. This is consistent with the recognition that DD species represent true research priorities (IUCN, 2001; Schipper et al., 2008; Butchart & Bird, 2010; Howard & Bickford, 2014; Bland et al., 2015; Luiz et al., 2016; Roberts, Taylor & Joppa, 2016; Jarić et al., 2016a).

## Materials & Methods

Latin names (including synonyms) of all species within the Kingdom Animalia classified as DD and CR were extracted from the IUCN Red List database (IUCN, 2015), as well as their years of assessment and scientific classification. The overall research effort directed towards each of the species was assessed within the Web of Science database (http://apps.webofknowledge.com, conducted during April–May 2016), by using their Latin names to search within titles, abstracts and keywords of articles published during 1996–2014. The assessment was focused on species classified within the given IUCN Red List categories during 2000–2010 only, in order to have a sufficient number of years before and after the classification to verify the output within the studied period. Some species were classified by the IUCN first as CR or DD, and then reclassified to another category. In such cases, these species were included in the sample only if they remained within CR or DD category at least four years after the original classification, and we limited the post-classification period only to those years. The analysis included only species described prior to the studied period (i.e., before 1996), to prevent the effects of species description interfering with the assessed trends.

We compared the observed patterns with those in species classified as Least Concern (LC), in which IUCN classification is not expected to produce a notable effect and which could therefore be considered as the baseline trend of publishing frequency over time. Research attention directed at LC species is related to other factors, such as their charisma, economic value, suitability for use as model species and accessibility, which makes them appropriate for use as a control group. Since the LC category comprised a substantial number of species (i.e., >10,000 species classified during 2000–2010; IUCN, 2015), their analysis was performed on a subsample that was obtained through stratified random sampling—namely, LC species were randomly included in the sample until each taxonomic subgroup (i.e., Arthropoda, Mammalia, etc.) reached the number of species that was equal to their numbers within the CR category.

There are different reasons for re-classification of species from one threat category to another, which may be either due to actual changes in conservation status (e.g., positive effects of conservation measures, increased threats) or due to other factors (e.g., changes in listing criteria, new information, changes in taxonomy). While we could not include such information in the species selection or in the analysis, we omitted the years in which DD or CR species were re-classified to other categories, as well as excluded all species that were reclassified between DD and CR categories, to avoid complex effects that such changes could produce on publishing rates.

### Method for detecting effects of IUCN listing

We test for the effect of IUCN listing on publication rate under the following statistical model. Consider a group of *J* species with the same listing category. We assume that over the observation period (0, *T*) publications on species *j* follow a Poisson process with rate function:

(1) \begin{eqnarray*}{\mu }_{j}(t)= \left\{ \begin{array}{@{}lll@{}} \displaystyle \vskip{-1pt}{\mu }_{j}&\displaystyle &\displaystyle 0\lt t\leq {\tau }_{j}\\ \displaystyle \beta {\mu }_{j}&\displaystyle &\displaystyle {\tau }_{j}\lt t\leq T \end{array} \right. \end{eqnarray*}

where *μ*_*j*_ is the unknown pre-listing publication rate for species *j*, *τ*_*j*_ is the known listing time for species *j*, and *β* is the unknown multiplicative listing effect that is assumed to be common for all species in the group. Under this model, the number *B*_*j*_ of publications prior to listing has a Poisson distribution with mean *μ*_*j*_*τ*_*j*_ and the number *A*_*j*_ of publications following listing has a Poisson distribution with mean *βμ*_*j*_ (*T* − *τ*_*j*_). It is a property of the Poisson distribution that, conditional on the total number *n*_*j*_ of publications during the observation period, *B*_*j*_ has a binomial distribution with *n*_*j*_ trials and success probability:

(2) \begin{eqnarray*}{p}_{j}= \frac{{\tau }_{j}}{{\tau }_{j}+\beta \left( T-{\tau }_{j} \right) } .\end{eqnarray*}

Inference about *β* can be based on the log likelihood function:

(3) \begin{eqnarray*}\log \nolimits L \left( \beta \right) =\sum _{j=1}^{J}{b}_{j}\log \nolimits {p}_{j}+ \left( {n}_{j}-{b}_{j} \right) \log \nolimits \left( 1-{p}_{j} \right) \end{eqnarray*}

where *b*_*j*_ is the observed value of *B*_*j*_. In particular, the maximum likelihood estimate $\hat {\beta }$ of *β* is found by maximizing Eq. (3) over *β*.

This model has two obvious limitations. First, it assumes that any publication effect occurs immediately after listing when, in reality, such an effect would appear only after a delay due to the time lag in funding application, research activity and publication. It is straightforward to extend the model to incorporate a common delay *δ* between the time of listing and the time at which the listing effect is manifested. We therefore included *δ* and compared the model output with and without it. As the results are insensitive to *δ*, for convenience, we present only those for the simpler model.

A more serious problem is that the model assumes that both the pre- and post-listing publication rates are constant. As a consequence, even in the absence of a listing effect, a steadily increasing publication rate would be reflected in a positive estimate of *β*. By the same token, a steadily decreasing publication rate could obscure a publication effect. To control for this, we based inference about a listing effect for DD and CR species groups on the differences:

(4) \begin{eqnarray*}{D}_{DD}& =& {\hat {\beta }}_{DD}-{\hat {\beta }}_{LC}\end{eqnarray*}

(5) \begin{eqnarray*}{D}_{CR}& =& {\hat {\beta }}_{CR}-{\hat {\beta }}_{LC}\end{eqnarray*}

where, for example, ${\hat {\beta }}_{DD}$ is the maximum likelihood estimate of *β* for species in the DD group. The idea here is that the LC group serves as a control, in the sense that ${\hat {\beta }}_{LC}$ reflects any common trend in publication rate independent of a listing effect (Larsen & Von Ins, 2010).

Briefly, we tested the null hypothesis of no DD listing effect against the one-sided alternative hypothesis of a positive listing effect by repeatedly randomizing the assignment of the pooled DD and LC species and re-calculating the value of *D*_*DD*_. The observed significance level (or *p* value) was approximated by the proportion of randomized data sets for which *D*_*DD*_ exceeded the observed value. An analogous procedure was used to test for a positive CR listing effect.

In the next step, we applied the method outlined above to species with at least one publication. Strictly speaking, this means that the statistical method should condition on this event. While this conditioning could create a significant technical problem, as the random variables *B*_*j*_ and *A*_*j*_ are no longer independent, the randomization remains a fully valid test.

## Results

**Figure 1:**
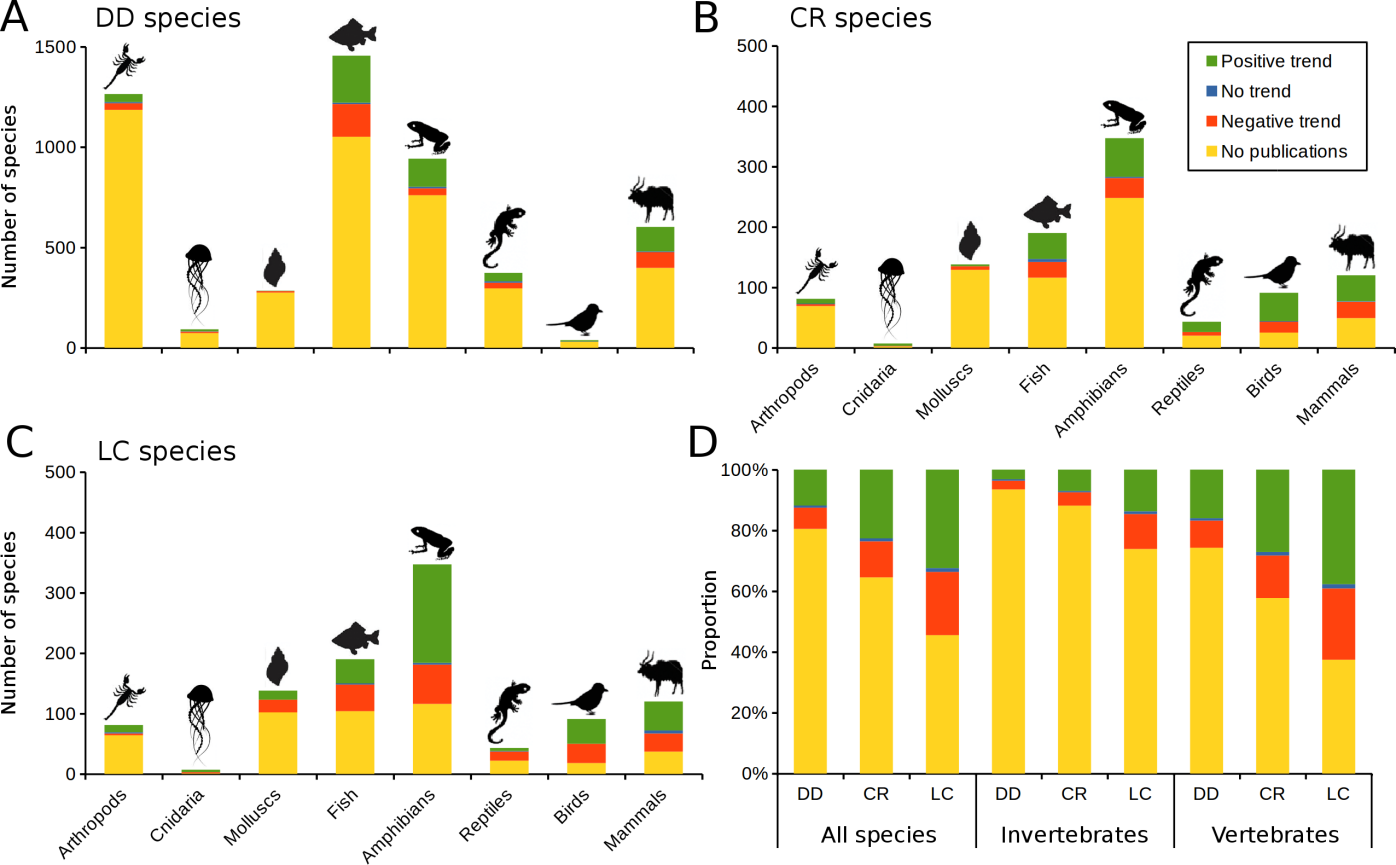
Influence of the IUCN Red List classification on research efforts. Number of species revealing one of the three trends (i.e., positive, neutral, negative) in publication output, based on the mean number of publications per year per species before and after their classification as DD, CR or LC on the IUCN Red list of threatened species (derived from Web of Science; http://apps.webofknowledge.com). Publication trends were adjusted for the general growth rate of scientific publication (Larsen & Von Ins, 2010).

Only a small proportion of CR and DD species appear to have benefited from an increasing publication output following their initial classification on the Red List, while the vast majority of species have not been addressed in any publication (Fig. 1). This was especially the case in DD species, and particularly within the group of invertebrates.

The statistical method presented here indicated presence of a listing effect in DD species (Table 1) in all assessed groups, except for invertebrates and mammals. The most expressed effect was observed in birds, amphibians and reptiles. At the same time, the listing effect observed in CR species was limited to reptiles and birds. Effect value (*β*) in LC species (Table 1) is considered to represent the baseline trend in publication rates. The three invertebrate species groups (arthropods, cnidarians and molluscs) had insufficient sample size due to too few species with publications, and therefore could not even be assessed here (Fig. 1).

**Table 1:** Maximum likelihood estimate of the DD and CR listing effect on publication rate (*β*) in different species groups, and the significance level (*p*). LC category represents the baseline publication trend with no listing effect; see the text for more information on the method.

Group	*β*			*p*	
	DD	CR	LC	DD	CR
All species	2.014	1.666	1.508	**0.002**	0.210
Vertebrates	1.998	1.655	1.530	**0.004**	0.272
Invertebrates	2.189	1.849	1.344	0.140	0.386
Fish	1.967	1.595	1.414	**0.018**	0.368
Amphibians	4.203	1.477	1.482	**0.000**	0.576
Reptiles	2.885	2.035	0.902	**0.000**	**0.006**
Birds	5.681	2.482	1.608	**0.000**	**0.000**
Mammals	1.849	1.669	1.902	0.544	0.782

## Discussion

In the present study, we tested for the effect of IUCN classification on publication rate in DD and CR species, two species groups generally recognized as research priorities (Brooks et al., 2008; De Lima, Bird & Barlow, 2011; Luiz et al., 2016; Roberts, Taylor & Joppa, 2016). We observed a significant listing effect in DD species, except for invertebrates and mammals. Research focus on DD species was however generally very low, with the majority of species receiving no publications during the studied period, and only a minor proportion of species experiencing positive trends in publishing rate (Fig. 1). In contrast to the DD listing, there was no effect of CR classification in the assessed species groups, except for birds and reptiles. The lack of listing effect we found here in mammals is in line with the findings of Brooke et al. (2014). On the other hand, the lack of listing effects in some CR species groups, especially in CR mammals, could also be caused by a high level of attention even before the listing, which perhaps made any further increase in research attention unlikely.

Most of the DD species were addressed in very few studies, in some cases even to the point that the entire species group could not be assessed. Nevertheless, results indicate that DD species, while chronically understudied, seem to be recognized by the scientific community as research priorities, and the IUCN listing seems to produce a desired, although arguably still insufficient effect in this species group. On the other hand, research effort for endangered species does not seem to be driven by the IUCN listing but by various other factors. These probably include species proximity to research institutions and wealthy nations, research costs and logistics demands influenced by species accessibility and range (Ficetola et al., 2013; Meyer et al., 2015). In plants, such patterns are also recognized as the “botanist effect” (Moerman & Estabrook, 2006; Pautasso & McKinney, 2007). Relevant factors also include economic importance or charisma of the species, funding policies, as well as the research inertia of the scientific community, which has a tendency to focus on the same, well-studied research models, on which expertise has been acquired, or which are already proven capable of attracting research funding (Martín-López, González & Montes, 2011; Jarić, Knežević-Jarić & Gessner, 2015). In conservation, research consequently remains focused on a small proportion of threatened species, while the majority receives little or no attention (Sitas, Baillie & Isaac, 2009; De Lima, Bird & Barlow, 2011). Such unbalanced research allocation results in a lack of information needed by policy makers and resource managers to develop sound conservation and restoration measures (Jarić, Knežević-Jarić & Gessner, 2015).

The method described in this paper for detecting the effect of listing on publication rate within a species group is based on the change in publication rate following listing. To control for any overall trend in publication rate, this change is compared to the corresponding change for species groups where no such change is expected (LC). Statistical significance is assessed using a randomization procedure that makes no assumption about the distribution of publication number. In other words, species can receive low overall research attention (i.e., manifested by a low number of publications per species), but still have a notable change in publication rate after listing, or conversely, they can be objects of intensive research, with a considerable number of publications, but without a significant change in publication rate after listing.

The Web of Science database is widely acknowledged as the most comprehensive and versatile tool for bibliometric analysis, with a representative publication sample to draw conclusions (Pyšek et al., 2008; Jarić, Knežević-Jarić & Gessner, 2015). In conservation research, assessment of trends and priorities often focuses on species as management units (Jarić et al., 2016b), and the species coverage in scientific publications and databases is considered as a good proxy to compare conservation attention over a large number of species (Sitas, Baillie & Isaac, 2009; Connena et al., 2017). As a result, similar approaches often have been applied within the field of conservation biology (e.g., Clark & May, 2002; Wilson et al., 2007; Trimble & Van Aarde, 2010; De Lima, Bird & Barlow, 2011; Robertson & McKenzie, 2015; Zhang et al., 2015; Donaldson et al., 2016). It is however important to note some drawbacks of Web of Science as a tool to evaluate scientific attention, mainly that it does not include grey literature, and is dominated by North American and European publications compared to other regions (Holmgren & Schnitzer, 2004; Pyšek et al., 2008; Haddaway & Bayliss, 2015; Jarić, Knežević-Jarić & Gessner, 2015). Nevertheless, even though a substantial amount of research effort ends up in grey literature, Web of Science database should still represent a suitable proxy of the overall research effort if one can assume that the publication output indexed by this database is proportional to the overall publication output per species. It is also important to note that we did not check for potential presence of geographic patterns, which could produce cofounding effects through spatial differences in scientific attention and publishing practices. Based on the results presented, it is apparent that there is a need to improve the current focus of research on biodiversity (Wilson et al., 2007). We argue for a need to develop international science planning efforts, to draw attention to the major gaps in the current knowledge. Awareness raising and increased support to this process should be facilitated through international mechanisms such as Convention on Biological Diversity (CBD) and the Intergovernmental Science-Policy Platform on Biodiversity and Ecosystem Services (IPBES). Sound species knowledge is especially relevant for the CBD Aichi Targets within the Strategic Plan for Biodiversity 2011–2020 (i.e., Target 19), with progress in target fulfilment assessed through indicators such as Species Status Information Index (GEO BON, 2015). Financial and other incentives should also be introduced for conservation oriented research and monitoring of both highly endangered and poorly studied species. International harmonization of funding programs with regard to their focus on closing knowledge gaps and the applicability for management measures would also contribute to this aim, especially if appropriate consideration of IUCN classification is ensured in the design of funding programs. In addition, incentives should be focused on local scientific capacity building in developing countries, where a plethora of both endangered and data-deficient species are located. Such prioritization of research focus would even be economically justified, as the law of diminishing returns suggests that the marginal increase in critical knowledge will be greater if research efforts are directed to least known species (De Lima, Bird & Barlow, 2011; Meyer et al., 2015). The scientific and funding communities therefore are facing the challenge to make better use of the IUCN Red List and rise to the challenge the IUCN data represent.

##  Supplemental Information

10.7717/peerj.4025/supp-1 Table S1 Dataset used for the analysis

## Additional Information and Declarations

### Data Availability

The following information was supplied regarding data availability: Jarić, Ivan (2017): Jarić et al., Science responses to IUCN Red Listing. Peerj - Supplementary material. figshare.
https://doi.org/10.6084/m9.figshare.5193583.v1.
